# Rationale and design of ASPIRE-ICU: a prospective cohort study on the incidence and predictors of *Staphylococcus aureus* and *Pseudomonas aeruginosa* pneumonia in the ICU

**DOI:** 10.1186/s12879-017-2739-4

**Published:** 2017-09-25

**Authors:** Fleur P. Paling, Darren P. R. Troeman, Martin Wolkewitz, Rubana Kalyani, Daniël R. Prins, Susanne Weber, Christine Lammens, Leen Timbermont, Herman Goossens, Surbhi Malhotra-Kumar, Frangiscos Sifakis, Marc J. M. Bonten, Jan A. J. W. Kluytmans

**Affiliations:** 1 Julius Center for Health Sciences and Primary Care, University Medical Center Utrecht, HP Stratenum 6.131, PO Box 85500, 3508 GA Utrecht, The Netherlands; 2 Institute for Medical Biometry and Statistics, Faculty of Medicine and Medical Center, University of Freiburg, Freiburg, Germany; 3 MedImmune, Gaithersburg, MD USA; 4 Laboratory of Medical Microbiology, Vaccine & Infectious Disease Institute (VAXINFECTIO), University of Antwerp, Wilrijk, Belgium; 5 AstraZeneca Pharmaceuticals LP, Gaithersburg, MD USA; 6 Department of Medical Microbiology, University Medical Center Utrecht, Utrecht, Netherlands; 7 Amphia Hospital, Breda, The Netherlands

**Keywords:** ICU pneumonia, *S. aureus*, *P. aeruginosa*, Vap

## Abstract

**Background:**

The epidemiology of ICU pneumonia caused by *Staphylococcus aureus* (*S. aureus)* and *Pseudomonas aeruginosa (P. aeruginosa)* is not fully described, but is urgently needed to support the development of effective interventions. The objective of this study is to estimate the incidence of *S. aureus* and *P. aeruginosa* ICU pneumonia and to assess its association with patient-related and contextual risk factors.

**Methods:**

ASPIRE-ICU is a prospective, observational, multi-center cohort study nested within routine surveillance among ICU patients in Europe describing the occurrence of *S. aureus* and *P. aeruginosa* ICU pneumonia. Two thousand (2000) study cohort subjects will be enrolled (50% *S. aureus* colonized) in which specimens and data will be collected. Study cohort subjects will be enrolled from a larger surveillance population, in which basic surveillance data is captured. The primary outcomes are the incidence of *S. aureus* ICU acquired pneumonia and the incidence of *P. aeruginosa* ICU acquired pneumonia through ICU stay.

The analysis will include advanced survival techniques (competing risks and multistate models) for each event separately as well as for the sub-distribution of ICU pneumonia to determine independent association of outcomes with risk factors.. A risk prediction model will be developed to quantify the risk for acquiring *S. aureus* or *P. aeruginosa* ICU pneumonia during ICU stay by using a composite score of independent risk factors.

**Discussion:**

The diagnosis of pathogen-specific ICU pneumonia is difficult, however, the criteria used in this study are objective and comparable to those in the literature.

**Trial registration:**

This study is registered on clinicaltrials.gov under identifier NCT02413242.

**Electronic supplementary material:**

The online version of this article (10.1186/s12879-017-2739-4) contains supplementary material, which is available to authorized users.

## Background

Patients hospitalized in the Intensive Care Unit (ICU) are at risk of acquiring pneumonia, especially if they are mechanically ventilated. Despite extensive efforts, ICU-acquired pneumonia continues to be one of the most frequently occurring complications in the ICU and increases morbidity as well as mortality [1, 2]. Accurately describing and predicting the occurrence of ICU pneumonia is difficult as a result of inconsistencies in the case definition and surveillance methods in different countries [1, 3]. The need for an accurate and standardized prognosis is important considering current interventions are preventive rather than therapeutic [4]. As part of the COMBACTE consortium (COMBatting AntibiotiC resisTance in Europe), two monoclonal antibodies (mAbs) are currently being developed that target *S. aureus* and *P. aeruginosa*, respectively [5, 6]. Both bacteria are frequently occurring causative pathogens of ICU pneumonia and administration of a mAb may prevent the development of ICU pneumonia with these pathogens [7, 8]. Prospective studies can assess possible risk factors that can identify subsets of patients that may benefit most from these interventions. Thus, the goal of this study is to systematically assess the impact of patient-related and contextual factors on the incidence of *S. aureus* and *P. aeruginosa* ICU pneumonia in Europe and to identify the patient subgroups that are at greater risk for disease and bear a disproportionate disease burden. These objectives will directly contribute to the sample size and feasibility calculations for clinical trial design of the mAb interventions [8].

## Methods

### Objectives

The primary objectives of this study are to determine the incidence of:

1. *S. aureus* ICU pneumonia through ICU stay; and
2. *P. aeruginosa* ICU pneumonia through ICU stay; and
  their independent associations with patient-related factors (e.g. colonization status, baseline serum antibody levels against *S. aureus* or *P. aeruginosa* antigens) and contextual factors.

The key secondary objectives are to develop risk prediction models to quantify the risk of acquiring (i) *S. aureus* or (ii) *P. aeruginosa* ICU pneumonia during ICU stay, by using a composite score of independent risk factors identified through primary objective 1 and 2. Other secondary and exploratory objectives can be found on ClinicalTrials.gov, under identifier NCT02413242, or in the online (Additional file 1: S.01).

### Study design

**ASPIRE-ICU** (**A**dvanced understanding of ***S*** *taphylococcus aureus* and **P**seudomonas aeruginosa **I**nfections in Eu**R**op**E** - **I**ntensive **C**are **U**nits) is a multi-center, prospective, observational cohort study nested within ongoing routine surveillance among ICU patients in Europe. The study is composed of two study populations, the surveillance population and study cohort population. The study cohort is nested within the larger surveillance population; this means that all data and specimens collected specifically for study cohort participants is *in addition* to data already captured by ways of surveillance. An overview of the schedule of procedures, including all sample collection types and time points can be found in the online (Additional file 1: Table S.02).

### Study populations and recruitment

#### Surveillance population

Patients eligible to participate in the surveillance population must be on mechanical ventilation (MV) upon or (expected to be) within 24 h after ICU admission and have an expected length of stay (LOS) of at least 48 h. Patients with an expected ICU stay of less than 48 h are at a lower risk for developing ICU infections since this population is generally healthier, without significant comorbidities and shorter in the ICU.

The surveillance population are considered to be the source population from which the study cohort subjects are derived. No informed consent is required for participation in the surveillance population, but depending on local legislation, patients of the surveillance population will receive information (i.e. leaflet/flyer) on the ASPIRE-ICU study and are able to deny use of their de-identified data for scientific purposes.

#### Study cohort population

Surveillance patients that meet the eligibility criteria described below for the study cohort population will be enrolled. 2000 study cohort subjects are required to meet the objectives of this study. Study cohort subjects are approached for informed consent based on their *S. aureus* colonization status at ICU admission. Subjects will be enrolled in a 1:1 ratio of *S. aureus* colonized subjects to non *S. aureus* colonized subjects with 1000 subjects in each stratum. A similar temporal distribution of enrolled *S. aureus* colonized and non *S. aureus* -colonized subjects will be managed by selecting the first non-colonized subject after including a colonized subject. For subjects unable to provide consent for any reason, a legally accepted representative may consent on the subject’s behalf at the time of enrollment.

Inclusion criteria for study cohort

1. Participant is 18 years or older at the time of enrollment.
2. Participant is on mechanical ventilation at ICU admission, or is (expected to be) within 24 h thereafter, based on investigator’s judgment.
3. Expected stay in ICU is 48 h or longer based on investigator’s judgment.
4. *S. aureus* colonization status is known within 72 h after start of first episode of mechanical ventilation and according to the result, the patient qualifies for enrollment.
5. Written informed consent from subject / legally accepted representative within 72 h after start of first episode of mechanical ventilation.

Exclusion criteria for study cohort

1. Previous participation as a subject in the study cohort of this study.
2. Simultaneous participation of the subject in any preventive experimental study into anti-staphylococcus or anti-pseudomonas aeruginosa interventions.
3. Expected death (moribund status) within 48 h, or ICU discharge of the participant within 24 h, at the moment of informed consent.

### Study outcome definitions

Considering that the definition of our primary outcome, ICU pneumonia caused by *S. aureus* or *P. aeruginosa,* is very extensive, we would like to refer to the (Additional file 1: S.03) containing the full definition. In summary, ICU pneumonia is defined as pneumonia occurring ≥48 h after admission to the ICU and is confirmed by a new or worsening infiltrate on chest X-ray or CT-thorax. Furthermore, the patient must fulfill specific clinical criteria (for example abnormal temperature, production of sputum, auscultatory abnormalities, acute changes in the ventilatory support system), in addition to at least 1 microbiological criterion (positive respiratory specimen, blood culture, pleural fluid aspirate or lung tissue culture).

### Site selection

In total, 30 sites in 11 countries were selected from sources including but not limited to the COMBACTE CLIN-Net and LAB-Net databases [7]. To ensure the pan-European continent is represented, there is at least one country included from the Northern / Southern / Eastern and Western region of Europe. The selected sites adequately balance different factors such as geography, background antibiotic resistance prevalence, etc.. To ensure enrollment is met within the expected timelines, back-up sites were selected for various reasons (i.e. low enrollment numbers, decline further participation) that can supplement or replace primary sites selected. For each site also a local laboratory was selected to participate in the study.

A Site Selection Committee selected sites and laboratories based on pre-defined criteria in the Site Selection Plan. Assessment of these criteria was aided by site feasibility questionnaires. Participating ICUs must have a routine *S. aureus* screening protocol in order to be selected for participation.

Screening should consist of a minimum of one nasal swab and one lower respiratory tract (LRT) sample analyzed locally on the day of ICU admission. The LRT sample is defined as the collection of an endotracheal aspirate (ETA) sample, or, if an ETA cannot be collected, a sputum sample may be taken. As an exception, for routine *S. aureus* colonization screening at ICU admission only, if both the ETA and sputum cannot be collected, a throat swab may be taken.

### Statistical analysis

#### Sample size calculation

Sample size calculations are based on the expected incidence precision of *S. aureus* ICU pneumonia and *P. aeruginosa* ICU pneumonia, since the primary objective of the study is to identify the patient groups most at risk for this outcome.

Assuming an incidence of *S. aureus* ICU pneumonia of 12.5% and 1.5% in the *S. aureus* colonized group and non *S. aureus* colonized group respectively, this would result in an overall incidence of 7% within the 2000 study cohort subjects, or 140 *S. aureus* ICU pneumonia endpoints [9]. The overall incidence estimate would have precision of 1.12% (95% confidence interval [CI]: 5.9%–8.12%, using normal approximation).

For *P. aeruginosa* ICU pneumonia, the overall incidence is estimated regardless of colonization status at ICU admission. Assuming an overall incidence of *P. aeruginosa* ICU pneumonia of 2.5% within the 2000 study cohort subjects, this would result in 50 *P. aeruginosa* ICU pneumonia endpoints. The overall incidence estimate would have precision of 0.68% (95% CI 1.82%–3.18%, using normal approximation).

#### Planned analysis

The primary analysis will evaluate the incidence density of *S. aureus* or *P. aeruginosa* ICU pneumonia; its calculation will depend on time from admission (for outcome ICU pneumonia) or time from ventilation (for outcome ventilator-associated pneumonia).

For the primary and secondary objectives, advanced survival techniques (competing risks and multistate models) will be applied. Discharge and death will be considered as competing events for ICU pneumonia. Adapted Cox regression models will be applied for each event separately as well as for the sub-distribution of ICU pneumonia. The clustering of the data (readmission, patients within ICU, country) will be acknowledged using shared frailty methodology, stratification or robust variance. The time-dependency of cumulative hazards and incidences will be graphically displayed, by risk factors of interest. Hazard ratios with 95% CI will be calculated univariately and selected for the multivariate model using an established Akaike’s information criterion for model selection. A risk prediction model will be developed to quantify the risk for acquiring *S. aureus* or *P. aeruginosa* ICU pneumonia during ICU stay by using a composite score of independent risk factors.

### Quality assurance

Data will be entered in a web-based electronic data capture system that was designed for **ASPIRE-ICU.** The study site will enter data in the electronic data capture system from the subject’s source documents (i.e. medical chart). Information linking the subject ID to the subject’s medical file (only applicable for study cohort subjects) will be kept in a secure place at the participating study site.

Monitoring will include 100% source data verification for the first three enrolled study cohort subjects at each site, and then 10% of the remaining enrolled study cohort subjects.

## Discussion

This manuscript describes the objectives and design of **ASPIRE-ICU**, an observational cohort study addressing risk factors for *S. aureus* and *P. aeruginosa* ICU pneumonia. Certain choices have been made in the design that warrant mention and further discussion.

### Definition of pathogen-specific pneumonia

The definition of *S. aureus* or *P. aeruginosa* ICU pneumonia is based on pre-defined pneumonia criteria in combination with the presence of the bacterium in an appropriate sample around the time of diagnosis, which are aligned with endpoints being used for two randomized controlled trials for prevention of *S. aureus* and *P. aeruginosa* pneumonia [8]. Thus, in case of cultures that yield multiple possible pathogens, it may be that the diagnosis of *S. aureus* or *P. aeruginosa* pneumonia is made incorrectly. The distinction between colonization and infection is sometimes difficult to assess. There is however no ‘reference standard’ to reliably assess the causative pathogen [3]. We have contemplated quantitative cultures in all pneumonia patients, but it was not feasible to implement this at each site. Quantitative measurements will however be applied on study samples received by the central laboratory, and thus will provide additional retrospective information. Furthermore, other outcomes, such as mortality will provide objective outcome information in addition to a diagnosis of pneumonia.

### Enrichment strategy

In this study, 50% of the study cohort population is *S. aureus* colonized at ICU admission to ‘enrich’ the study population with *S. aureus* carriers (in nose or lower respiratory tract), while this naturally occurs in approximately 20–25% of the ICU population [10]. This was chosen as *S. aureus* carriage is being studied as one of the main known risk factors for subsequent *S. aureus* disease, thus without enrichment, the population needed for equal precision would be much larger [9, 11, 12]. However, the limitation of this choice is that one can argue that a population as such is not representative of the general ICU population, thus incidence estimation as well as assessing risk factors for *P. aeruginosa* ICU pneumonia may be suboptimal. We acknowledge this, and for this reason the surveillance population was included. Their data will allow distribution of baseline factors with the study cohort in an effort to assess the ubiquity of results across both groups. Furthermore, it can serve to identify potential bias between participants and non-participants. Considering that *P. aeruginosa* colonization at ICU admission is relatively rare and that colonization often occurs after ICU admission, no recruitment selection for *P. aeruginosa* colonized subjects will take place [13].

### Routine surveillance

This study utilizes routine *S. aureus* screening conducted at each participating site as the basis for eligibility assessment. For consistency of screening results across sites, all swabs will be plated under protocol on the same chromogenic agar plates (Colorex agar, bioTRADING Benelux) provided by the ASPIRE-ICU study team. In the original protocol, a nose swab and ETA sample (sputum sample if non-intubated) are collected at screening. However, soon after study start, the protocol was amended to include a throat sample as a LRT sample, in case ETA and sputum were both not feasible.

### Future perspectives

In this era of increasing antimicrobial resistance, the research field is steadily exploring other therapies, for example prophylactic therapies such as antibody-based preventive measures. A potential advantage of monoclonal antibodies (mAbs) is that they will not encourage bacterial resistance to the same extent as antibiotics, and may even augment antibiotic effectiveness [14]. This study was designed in part to provide crucial information on the incidence, patient-related and contextual factors of ICU pneumonia caused by *S. aureus* and *P. aeruginosa*, but also inform the design of future phase III trials, that will investigate mAbs effectiveness against *S. aureus* and *P. aeruginosa*. Two large COMBACTE phase II trials, SAATELLITE (A Human Monoclonal Antibody Against *Staphylococcus aureus* Alpha Toxin in Mechanically Ventilated Adult Subjects) and EVADE (Effort to Prevent Nosocomial Pneumonia Caused by *Pseudomonas aeruginosa* in Mechanically Ventilated Subjects) have already started [8]. SAATELLITE investigates the effect of MEDI4893, a mAb targeting *S. aureus* alpha toxin and EVADE studies MEDI3902, which is another mAb that simultaneously targets PcrV and Psl on the surface of the *P. aeruginosa* bacterium, in subjects at risk for ICU pneumonia [5, 6, 8]. These targeted therapies, if proven effective, may be used in the future for patients at highest risk. This study will help to identify the subset of patients that will likely benefit most.

## Conclusion

This epidemiological cohort study on *S. aureus* and *P. aeruginosa* ICU pneumonia aims to add significant information to the literature on predictors for this event. This will help refine the design of Phase III trials and may benefit patients at risk for nosocomial infections, by providing protective measures.

## Additional file

Additional file 1: S.01. Complete list of objectives and endpoints. **Table S 02.** Schedule of procedures. **S.03.** Complete definition of study endpoints. (DOCX 29 kb)

## Abbreviations

ASPIRE-ICU: Advanced understanding of *Staphylococcus aureus* and Pseudomonas aeruginosa Infections in EuRopE
CI: Confidence interval
COMBACTE: COMBatting AntibiotiC resisTance in Europe
ETA: Endotracheal aspirate
ICU: Intensive Care Unit
LOS: Length of stay
LRT: Lower respiratory tract
mAb: Lower respiratory tract
*P. aeruginosa*: *Pseudomonas aeruginosa*
*S. aureus*: *Staphylococcus aureus*
