# Supplementary material for: Rationale and design of ASPIRE-ICU: a prospective cohort study on the incidence and predictors of Staphylococcus aureus and Pseudomonas aeruginosa pneumonia in the ICU
Source: BMC Infect Dis. 2017 Sep 25;17:643. doi: 10.1186/s12879-017-2739-4 (PMC5613521; doi:10.1186/s12879-017-2739-4)
Supplement: Additional file 1: S.01. — Complete list of objectives and endpoints. Table S 02. Schedule of procedures. S.03. Complete definition of study endpoints. (DOCX 29 kb) [file 12879_2017_2739_MOESM1_ESM.docx]

Supplemental material

S.01Complete list of objectives and endpoints

*Objectives*

Primary objectives

1. To determine the incidence of ICU pneumonia caused by *S. aureus* through ICU stay and its independent association with patient-related factors (e.g. baseline serum antibody levels against *S. aureus* alpha toxin [binding and functional], *S. aureus* colonization in nose/ETA) and contextual factors.
2. To determine the incidence of ICU pneumonia caused by *P. aeruginosa* through ICU stay and its independent association with patient-related factors (e.g. baseline *P. aeruginosa* serum antibody levels against Psl and PcrV [binding and functional], *P. aeruginosa* colonization in peri-anal region/ETA), and contextual factors.

Secondary objectives

1. To develop a risk prediction model to quantify the risk of acquiring *S. aureus* ICU pneumonia during ICU stay, by using a composite score of independent risk factors identified through primary objective 1.
2. To develop a risk prediction model to quantify the risk of acquiring *P. aeruginosa* ICU pneumonia during ICU stay, by using a composite score of independent risk factors identified through primary objective 2.
3. Diagnostic
   1. To determine if a rapid, PCR-based diagnostic is as sensitive and specific as traditional culture to identify *S. aureus* colonization .
   2. To determine if a rapid, PCR-based diagnostic is as sensitive and specific as traditional culture to identify *P. aeruginosa* colonization.
4. To determine the incidence of all-cause ICU pneumonia (and VAP specifically) and to describe its temporal distribution in relation to hospitalization in the ICU.
5. To assess the incidence of ICU pneumonia attributable to *S. aureus* stratified by methicillin susceptibility (methicillin-susceptible *S. aureus* [MSSA] and methicillin-resistant *S. aureus* [MRSA]).
6. To assess the incidence of ICU pneumonia attributable to *P. aeruginosa* stratified by multi-drug-resistance (multi-drug resistant *P. aeruginosa* [MDR-PA] and susceptible *P. aeruginosa* [S-PA]).
7. To assess the incidence of ICU acquired bacteremia by etiologic agent (*S. aureus*, *P. aeruginosa* and/or for all other clinically relevant other pathogens combined) and to describe its temporal distribution in relation to hospitalization in the ICU.
8. To assess the independent association of *S. aureus* nasal colonization with all-cause mortality and risk of *S. aureus* infection.
9. To assess the independent association of *P. aeruginosa* peri-anal colonization with all-cause mortality and risk of *P. aeruginosa* infection.
10. To assess expression of known (AT, ClfA, SpA, ISDH etc.) and other virulence factors, as identified by transcriptomics/proteomics experiments (toxinome studies) in *S. aureus* isolates associated with colonization or ICU pneumonia.
11. To assess the gene sequence of *S. aureus* known virulence factors (AT, ClfA, SpA, ISDH etc.) and of those identified in toxinome studies in *S. aureus* isolates associated with colonization or ICU pneumonia.
12. To assess *P. aeruginosa* isolates associated with colonization or ICU pneumonia for variations in PcrV, Psl, and associated genes.
13. To assess PcrV, Psl, and other virulence factors expression under anti-infective pressure in *in* *vitro* biofilm model and in VAP and pneumonia animal models.
14. Biomarkers:
    1. To explore the role of antibodies against *S. aureus* virulence factors (for example *clumping* factor A [ClfA], Staphylococcal protein A [SpA], and [ISDH] and those identified in toxinome studies) as potential biomarkers associated with *S. aureus* infection.
    2. To assess the independent association between baseline serum antibody levels against the *Pseudomonas aeruginosa* PcrV and polysaccharide synthesis locus (Psl) virulence factors and *P. aeruginosa* infection.
    3. To assess the independent association between host biomarkers (e.g. baseline antibody levels against pathogen virulence factors, inflammatory markers, differentially expressed RNA molecules and proteins), the occurrence of ICU pneumonia and clinical outcomes among cases of ICU pneumonia.
    4. To assess the independent association between pathogen biomarkers (e.g. presence of *P. aeruginosa* or *S. aureus* virulence factors) and clinical outcomes among cases of *P. aeruginosa* or *S. aureus* infections.

Exploratory objectives

1. To describe magnitude of healthcare utilization associated with *S. aureus* ICU pneumonia (e.g. length of ICU stay and duration of mechanical ventilation).
2. To describe magnitude of healthcare utilization associated with *P. aeruginosa* ICU pneumonia (e.g. length of ICU stay and duration of mechanical ventilation).
3. To determine strain characteristics of *S. aureus* and *P. aeruginosa* isolates from ICU pneumonia cases
   1. Determinants of resistance, virulence and other relevant genes
   2. To assess the proportion of *S. aureus* isolates in which the AT-gene is present on the genome.
   3. Monitor prevalence of *S. aureus* clonal types associated with colonization and ICU pneumonia cases.
   4. To assess the proportion of *P. aeruginosa* isolates on which the *Pseudomonas aeruginosa* PcrV or polysaccharide synthesis locus (Psl) gene is present.
4. To explore the role of antibodies against Gram-positive and Gram-negative bacterial virulence factors as biomarkers.
5. To identify independent risk factors for acquiring *S. aureus* colonization during ICU stay.
6. To identify independent risk factors for acquiring *P. aeruginosa* colonization during ICU stay.
7. To compare participating study sites and sites participating in routine HAI surveillance (e.g., ECDC HAI-Net or other external data sources) to further inform external validity of results.

*Endpoints*

Primary endpoints

1. Incidence of *S. aureus* ICU pneumonia in subjects until ICU discharge.
2. Incidence of *P. aeruginosa* ICU pneumonia in subjects until ICU discharge.

Secondary endpoints

1. Prevalence of *S. aureus* / *P. aeruginosa* colonization at ICU admission in subjects.
2. Incidence of all cause ICU pneumonia in subjects until ICU discharge.
3. Incidence of *S. aureus* ICU pneumonia stratified by MRSA vs. MSSA.
4. Incidence of *P. aeruginosa* ICU pneumonia stratified by MDR-PA vs. S-PA.
5. Incidence of ICU bacteremia per etiologic agent (in case of *S. aureus* and/or *P. aeruginosa* and for all clinically relevant other pathogens) in subjects until ICU discharge.
6. All-cause mortality throughout ICU stay.
7. All-cause mortality at day 30 after ICU admission.
8. All-cause mortality at day 90 after ICU admission.
9. Time to *S. aureus* ICU pneumonia until ICU discharge.
10. Time to *P. aeruginosa* ICU pneumonia until ICU discharge.
11. Time to all cause ICU pneumonia until ICU discharge.
12. Time to all cause ICU bacteremia until ICU discharge.
13. Time to death of any cause up to 90 days following ICU admission or until ICU discharge.

Exploratory endpoints

1. Magnitude of healthcare utilization as measured by:
   1. Duration of ICU stay including readmissions
   2. Days on mechanical ventilation
   3. Days of antibiotic usage
   4. Duration of hospital stay, including readmissions
2. Incidence of *S. aureus* colonization after ICU admission but prior to ICU pneumonia.
3. Incidence of *P. aeruginosa* colonization after ICU admission but prior to ICU pneumonia.

| **Table S.02 Schedule of procedures** | | | | | | | | | | | |
| --- | --- | --- | --- | --- | --- | --- | --- | --- | --- | --- | --- |
| *Procedure* | **All eligible patients** | **All enrolled study cohort subjects** | | | | | | | **Additional procedures for each ICU-pneumonia** | | |
|  | Day of ICU Admission | D1 | D4 | D7 | D8-D30 | D30 or ICU discharge | Across ICU stay until discharge | | Day of ICU-pneu | D7 after ICU-pneu or ICU discharge | D30 after ICU-pneu or ICU discharge |
| Verify eligibility criteria | X |  |  |  |  |  |  | |  |  |  |
| ICU information flyer (including information of ongoing study) | X |  |  |  |  |  |  | |  |  |  |
| Assignment of SID number | X |  |  |  |  |  |  | |  |  |  |
| Collection of anonymized surveillance data | X |  |  |  |  |  | X | |  |  |  |
| Nasal swab sample for SA colonization status | X ^a^ |  |  |  |  |  |  | |  |  |  |
| Lower respiratory tract sample^b^ for SA colonization status | X ^a^ |  |  |  |  |  |  | |  |  |  |
| Additional study procedures for subjects **enrolled** in the study cohort | | | | | | | | | | | |
| Written informed consent |  | X |  |  |  |  |  | |  |  |  |
| Collection of additional data from medical charts |  | X ^c^ |  | X ^c^ |  |  | X | | X ^c^ |  |  |
| Assessment of infection status |  |  |  |  |  |  | X | |  |  |  |
| Assessment of mortality status |  |  |  |  |  | X | X ^d^ | |  |  |  |
| Nasal swab sample for SA colonization status |  | X ^e^ | X ^e^ | X ^e^ |  |  |  | |  |  |  |
| Peri-anal sample for PA colonization status |  | X ^e^ | X ^e^ | X ^e^ | X ^e, f^ |  | |  |  |  |  |
| Lower respiratory tract sample^b^ for SA/PA colonization status and biomarkers |  | X ^e^ | X ^e^ | X ^e^ | X ^e, f^ |  | |  |  |  |  |
| BAL sample for PA colonization status |  |  |  |  |  |  | | X ^e, g^ |  |  |  |
| Blood (serum) sample to assess SA anti-AT, PA anti-Psl and anti-PcrV antibody levels and humoral immunomics |  | X ^e^ |  | X ^e^ |  | X ^e^ | |  | X ^e, h^ | X ^e^ | X ^e^ |
| *Procedure* | **All eligible patients** | **All enrolled study cohort subjects** | | | | | | | **Additional procedures for each ICU-pneumonia** | | |
|  | Day of ICU Admission | D1 | D4 | D7 | D8-D30 | D30 or ICU discharge | | Across ICU stay until discharge | Day of ICU-pneu | D7 after ICU-pneu or ICU discharge | D30 after ICU-pneu or ICU discharge |
| Blood (EDTA) sample for host protein biomarkers |  | X ^e^ |  | X ^e^ |  |  | |  | X ^e, h^ |  |  |
| Whole blood sample for RNA biomarkers |  | X ^e^ |  | X ^e^ |  |  | |  | X ^e, h^ |  |  |
| Lower respiratory tract sample^b^ to assess SA/PA infection status |  |  |  |  |  |  | |  | X ^e, h^ | X ^e^ |  |
| Medication use (antibiotics / immunosuppressive medication) |  | X |  |  |  |  | | X | X |  |  |

S.03 Complete definition of study endpoints

- 1. ICU pneumonia in mechanically ventilated patients

Patient should demonstrate the following new onset of symptoms/signs deemed not due to any overt non‑infectious causes.

**a. Radiographic criteria:**

New or worsening infiltrate consistent with pneumonia on chest X-ray or CT-thorax obtained within 24 hours of the event (diagnosed by a qualified radiologist).

**AND**

**b. Clinical criteria:**

At least **2** of the following minor or **1** major respiratory sign or symptom of new onset:

Minor criteria:

- Systemic signs of infection (one or more of the following): Abnormal temperature (oral or tympanic temperature > 38°C or a core temperature ≥ 38.3°C or hypothermia, defined as a core body temperature of < 35°C), and/or abnormal WBC (WBC count > 10,000 cells/mm^3^, WBC count < 4500 cells/mm^3^, or > 15% band neutrophils)
- Production of purulent endotracheal secretions
- Auscultatory findings consistent with pneumonia/pulmonary consolidation (e.g. rales, rhonchi, bronchial breath sounds, dullness to percussion)

Major criteria: Acute changes made in the ventilatory support system to enhance oxygenation, as determined by:

- PaO_2_/FiO_2_ ratio < 240 mmHg, or
- A decrease in PaO_2_/FiO_2_ by ≥ 50 mmHg
  1. ICU pneumonia in not mechanically ventilated patients

Patient should demonstrate the following new onset of symptoms/signs deemed not due to any overt non‑infectious causes.

**a. Radiographic criteria:**

New or worsening infiltrate consistent with pneumonia on chest X-ray or CT-thorax obtained within 24 hours of the event (diagnosed by qualified radiologist)

**AND**

**b. Clinical criteria:**

At least **2** of the following minor or **1** major respiratory signs or symptoms:

Minor criteria:

- Systemic signs of infection: Abnormal temperature (oral or tympanic temperature > 38°C or a core temperature ≥ 38.3°C or hypothermia, defined as a core body temperature of < 35°C), and/or abnormal WBC (WBC count > 10,000 cells/mm^3^, WBC count < 4500 cells/mm^3^, or > 15% band neutrophils)
- A new onset of cough (or worsening of cough)
- Production of purulent sputum
- Physical examination findings consistent with pneumonia/pulmonary consolidation such as auscultatory findings (e.g. rales, rhonchi, bronchial breath sounds), dullness to percussion, or pleuritic chest pain
- Dyspnea, tachypnea (respiratory rate > 30 breaths/minute), or hypoxemia defined as:
  - O_2_ saturation < 90% or PaO_2_ < 60 mmHg on room air if lower than baseline, or
  - A need to initiate or increase sustained (≥ 3 hours) supplemental oxygen to maintain pre‑event baseline O_2_ saturations

Major criteria:

A need to initiate non-invasive mechanical ventilation or re-initiate invasive mechanical ventilation because of respiratory failure or worsening of respiratory status

- 1. *S. aureus* ICU pneumonia in mechanically ventilated patients

Patient should meet all criteria as described for ICU pneumonia in mechanically ventilated patients (S.03.1) **AND** at least 1 of the following microbiological criteria:

- Respiratory specimen (obtained within 72 hours of onset of the event) is positive for *S. aureus* by culture. Includes a specimen of respiratory secretions obtained by endotracheal aspiration or by bronchoscopy with bronchoalveolar lavage (BAL) or protected-specimen brush (PSB) sampling in intubated subjects
- Blood culture positive for *S. aureus* (and no apparent primary source of infection outside the lung)
- Pleural fluid aspirate or lung tissue culture positive for *S. aureus* during episode of pneumonia (only if obtained as part of the subject’s necessary clinical management or post-mortem)
  1. *S. aureus* ICU pneumonia in not mechanically ventilated patients

Patient should meet all criteria as described for ICU pneumonia in not mechanically ventilated patients (S.03.2) **AND** at least 1 of the following microbiological criteria:

- Respiratory specimen (obtained within 72 hours of onset of the event) is positive for *S. aureus* by culture. Includes either expectorated sputum or (only if obtained as part of the subject’s necessary clinical management or post-mortem) a specimen of respiratory secretions obtained by bronchoscopy with BAL or PSB sampling. Respiratory samples from expectoration must show < 10 squamous epithelial cells and > 25 polymorphonuclear neutrophils per 100x field to be suitable.
- Blood culture positive for *S. aureus* (and no other apparent primary source of infection outside the lung)
- Pleural fluid aspirate or lung tissue culture positive for *S. aureus* (only if obtained as part of the subject’s necessary clinical management or post-mortem)
  1. *P. aeruginosa* ICU pneumonia in not mechanically ventilated patients

See S.01.3 but replace *S. aureus* with *P. aeruginosa*.

- 1. *P. aeruginosa* ICU pneumonia in not mechanically ventilated patients

See S.01.4 but replace *S. aureus* with *P. aeruginosa*.
